# Embodied cognitive intelligence guided Moon sample collection

**DOI:** 10.1016/j.xinn.2025.100939

**Published:** 2025-04-29

**Authors:** Chunjie Zhang, Chuankai Liu, Shaohua Duan, Xiaolong Zheng, Tianyi Yu, Jitao Zhang

**Affiliations:** 1 Institute of Information Science, School of Computer Science and Technology, Beijing Jiaotong University, Beijing 100044, China; 2 Visual Intellgence+X International Cooperation Joint Laboratory of MOE, School of Computer Science and Technology, Beijing Jiaotong University, Beijing 100044, China; 3 Beijing Aerospace Control Center, Beijing 100190, China; 4 State Key Laboratory of Multimodal Artificial Intelligence Systems, Institute of Automation, Chinese Academy of Sciences, Beijing 100190, China; 5 School of Artificial Intelligence, University of Chinese Academy of Sciences, Beijing 100190, China

## Main text

The success of Chang’e-6^1^^,^^2^ is a milestone of lunar exploration by China, being the first successful attempt in human history to collect samples from the far side of the Moon. To ensure collection efficiency and success of the mission, operators on the Earth needed to cooperate with the sample collection equipment on the Moon and finish the sample collection in less than a day. Embodied cognitive intelligence (ECI), which dynamically combines the advantage of human cognition with embodied intelligence, fitted this mission well. Experts on the Earth accumulated experience and knowledge to guide the design of the Chang’e probe and also conducted extensive simulation experiments for various challenges arising on the Moon. We used these accumulated data and experience along with knowledge to construct a cognitive map and then use the real-time data of Chang’e-6 in an embodied way by intelligently interacting with the environment. By using ECI, we successfully collected samples on the far side of the Moon in a fast, accurate, and robust way.

## Moon’s exploration by China

In 2013 Chang’e-3, equipped with a robotic arm, landed on the Moon. In 2018–2019, Chang’e-4 landed on the far side of the Moon. In 2020, the Chang’e-5 probe collected 1,731 g of sample on the near side of the Moon. In 2024, Chang’e-6 was launched and collected 1,935.3 g of sample on the far side of the Moon. The Chang’e-7 and Chang’e-8 probes will be launched in the next few years. An International Lunar Research Station (ILRS) has also been planned for long-term lunar exploration.

## Embodied cognitive intelligence

The long-term study of mammals indicates the usefulness of cognitive mapping^3^ for environment cognition, interpretation, and adaptation. The interaction between brain and environment provides rich experience and basic knowledge for cognition. Cognitive mapping can gradually adapt to the changing environment. Embodied intelligence^4^ tries to integrate physical interaction with artificial intelligence (AI) in real-world scenarios, which is a possible path to artificial general intelligence. It has shown potential in various applications (e.g., robotics, autonomous driving, and intelligent manufacturing). However, it also faces shortcomings such as the requirement of rich data and high computational cost. Besides, its performance is still unsatisfactory for high-cost and dangerous applications (e.g., lunar exploration and toxic chemical waste disposal). Human experience and knowledge should additionally be used.

**Figure 1:**
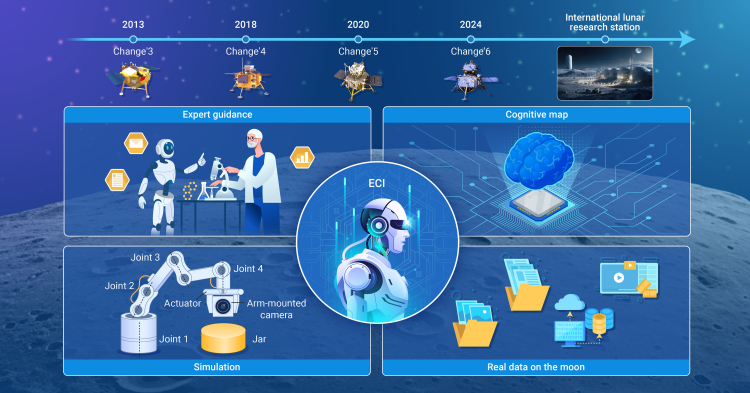
Embodied cognitive intelligence helps surface sample collection on the Moon

We introduced an ECI framework to jointly combine simulated and real operational data, an AI model, and human cognition to accomplish surface sample collection on the Moon, as shown in Figure 1. First, an initial cognitive map is constructed with both simulated and historical data, along with the rich experience and knowledge of human experts. The initial cognitive map can cope with basic operations and decision-making requirements. Using the cognitive map, real data can then be used along with the AI model to cope with different circumstances on the Moon. The cognitive map is then renewed with human supervision to provide reliable updates. This process is iterated and can consistently improve the performance. By constructing a task-oriented cognitive map along with a well-designed evolving scheme, the ECI framework can be used for various applications with efficient, reliable, and continually improved performance.

## Moon sample collection using ECI

Moon sample collection includes drill-down sample collection and surface sample collection. The drill-down operation of Chang’e-5 and Chang’e-6 only reached approximately 1 m. Nevertheless, surface sample collection can collect relatively more sample and has become the main method of collection. However, simulated experiments on the Earth showed that there were two challenges to reliable sample collection for Chang’e-6. (1) Limited sample collection time: Chang’e-6 has only approximately half the collection time of Chang’e-5 because communication can be interrupted when collecting sample on the far side of the Moon. (2) Unpredictable illumination conditions: the key points for sample placement may not be accurately detected due to poor and varying illumination of Chang’e-6. This requires carrying out the sample collection in a fast, accurate, and robust way.

ECI was able to cope well with the sample-collecting mission of Chang’e-6 by targeting these two challenges. We accumulated knowledge to guide the design of the probe before sending each Chang’e probe to the Moon. Extensive simulation experiments were also carried out to cope with the various challenges anticipated to be faced on the Moon. To speed up the sample collection process, we construct a cognitive map for basic operation control and update it over the missions. Serial operations are intelligently combined with optimized instructions. In this way, Chang’e-5 helped to improve the operational efficiency of Chang’e-6, which can then be used for the Chang’e-7 and Chang’e-8 missions in the future. The cognitive map jointly models simulated data, expert instruction, operational status, and real data on the Moon. To cope with illumination influences, we analyze the real-time visual data (captured by observation and hand-eye cameras) for sample collection with intelligent key-point and sample container edge detection, while 3D information from Chang’e-5 and Chang’e-6 is also used for detection and cross-validation. Under extreme conditions when no key point can be detected, expert instructors manually label a few key points for assistance. In this way, ECI can accurately collect and place the samples into the sample container several times until a sufficient amount is collected.

## Post-verification of Chang’e-5

The surface sample collection of Chang’e-5 was conducted in a remote-control manner. Accurately locating the sample container is the key to the success of sample collection, which should be controlled at millimeter scale. Each manual sampling operation takes approximately 1.5 h on average. The whole task takes more than 20 h. Several checkerboards are used for accurate sample placement by manual labeling. The historical data of Chang’e-3 and Chang’e-4 are used to generate a cognitive map with 3D information supervision for basic sample collection, and the data of Chang’e-5 are used to update the cognitive map in an embodied way. Simulated collection experiments on historical data of Chang’e-5 show that each sampling operation time can be improved from approximately 1.5 h to minutes.

## Practical application of Chang’e-6

Intelligent surface sampling collection and placement is the key to success of the Chang’e-6 mission. A Moon-touching disk is used to assist in sample collection and avoid equipment damage. An embodied intelligent ellipse detection method is used to detect the Moon-touching disk, which provides essential support for subsequent surface sampling operations. Compared with Chang’e-5, each sampling time of Chang’e-6 is reduced to approximately one-half to one-third that of Chang’e-5 on average. The sampling time of Chang’e-6 is reduced to approximately 11 h. The time per shoveling operation is reduced from 1.5 h to 0.5 h. In addition, collection efficiency is also improved. Chang’e-6 collects 1,935.3 g sample with a reduction of 33% shoveling operations compared to Chang’e-5. On average, 144.25 g per shoveling operation is made by Chang’e-5, while this amount is increased to 241.9125 g per shoveling operation by Chang’e-6. Moreover, the illumination conditions of Chang’e-6 are more complex than those of Chang’e-5, as we are also able to tackle the illumination variations of Chang’e-6 with ECI.

## Future viability of Chang’e-7, Chang’e-8, and ILRS

In the next few years, China will send Chang’e-7 and 8 to carry out more scientific explorations of the Moon. The surface sample is one of the most important aspects to be studied. As some of the loads are movable, collecting and analyzing the surface sample for different loads is a challenging problem. The ILRS also requires a thorough exploration of the geological conditions on the Moon.^5^ Moreover, the drilling operations of Chang’e-5 and Chang’e-6 only reached approximately 1 m, much shorter than expected. This highlights the complex geological conditions on the Moon that will be encountered in the upcoming missions. As discussed by Zhao et al.,^5^ geological experts can greatly advance the development of geoscience by leveraging AI models along with expert knowledge. ECI is a promising way for geological exploration of the Moon with accumulated expert experience, knowledge, and geological data. Exploration of the geological conditions with ECI will pave the way for automated analysis, prediction, and control of the lunar probes and loads for space exploration by China and the ILRS.

## Funding and acknowledgments

This work was supported by the 10.13039/501100001809National Natural Science Foundation of China (grant nos. 62476021, 72225011, 72434005, 62373034, and 62072026).

## Declaration of interests

The authors declare no competing interests.
